# Supplementary material for: Spatial Patterning of Fluorescent Liquid Crystal Ink Based on Inkjet Printing
Source: Molecules. 2022 Aug 28;27(17):5536. doi: 10.3390/molecules27175536 (PMC9458137; doi:10.3390/molecules27175536)
Supplement: Supplementary file 1 [file molecules-27-05536-s001.zip › molecules-1854241-supplementary.pdf]

SUPPORTING INFORMATION

Spatial Patterning of Fluorescent Liquid Crystal Ink Based on Inkjet Printing

Lei Zhang, Yongfeng Cui, Qi Wang, Huimin Zhou, Hao Wang, Yuzhan Li, Zhou Yang, Hui Cao, Dong Wang and Wanli He *

School of Materials Science and Engineering, University of Science and Technology Beijing, Beijing 100083, China
* Correspondence: hewanli@mater.ustb.edu.cn; Tel.: +86-13522735763

.Synthesis

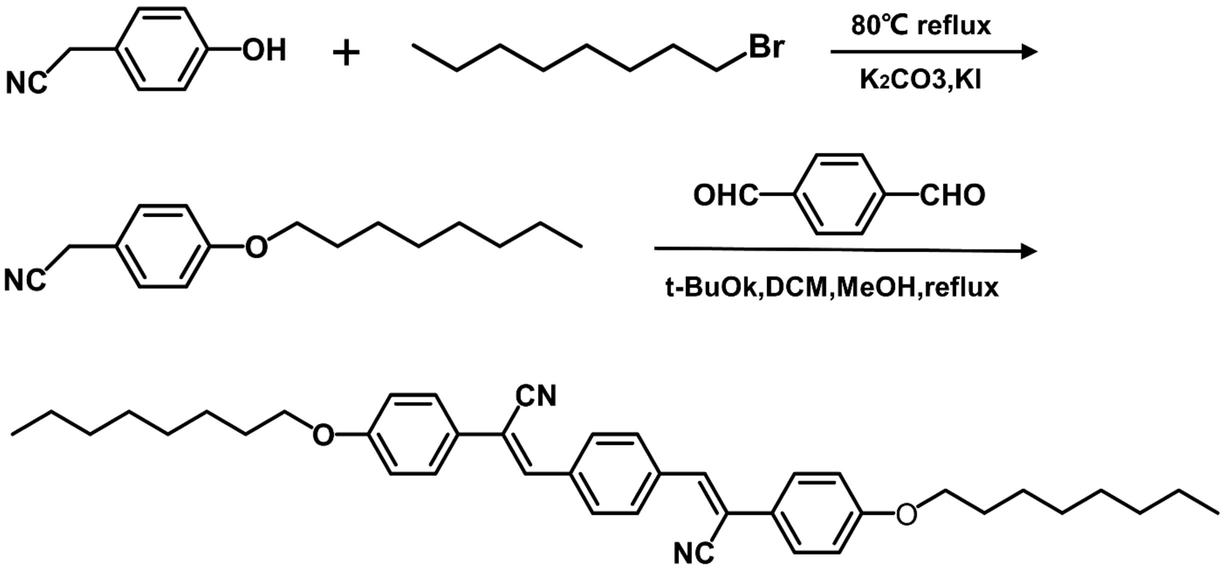

Figure S1. Synthetic route of fluorescent molecule DC8

4-cyanomethylphenol (2.0 g, 15 mmol), 1-bromooctane (3.0 g, 15. 5 mmol), K_2CO_3 (1.6 g, 15.5 mmol) and potassium iodide were added in 2-butanone (40 mL). The mixture was heated to 83 °C for 8 h and then washed with deionized water. After extraction with dichloromethane, the combined organic layers were dried over Na_2SO_4 and concentrated. The crude product was purified by column chromatography (DCM/PE, 4:1) on silica gel, which is a yellow transparent liquid (3.5 g, 70%). The intermediate is used as a reactant for the next reaction step. The intermediate (3.0 g, 14.46 mmol) and terephthalaldehyde (1.0 g, 7.4 mmol) was dissolved in dichloromethane (30 mL). The mixture was heated to 50 °C. A solution of potassium tert-butoxide (1.6 g, 14.8mmol) in methanol (20 mL) was added dropwise with stirring. After the reaction was completed, the crude product was purified by column chromatography with dichloromethane on silica gel. After concentration and drying, a yellow

solid was obtained (2.5g, 62%).^{1}H NMR (600 MHz, DMSO-d6) δ 7.86 (s, 2.22H), δ 7.80 (d, J = 2.1 Hz, 4.31H), 7.32 (d, J = 2.2 Hz, 4.15H), 6.99 (d, J = 6.5 Hz, 4.22H), 4.04 (s, 4H), 1.67 (d, J = 6.9 Hz, 5.14H), 1.43 – 1.27 (m, 17H), 0.89 – 0.86 (m, 7.22H).

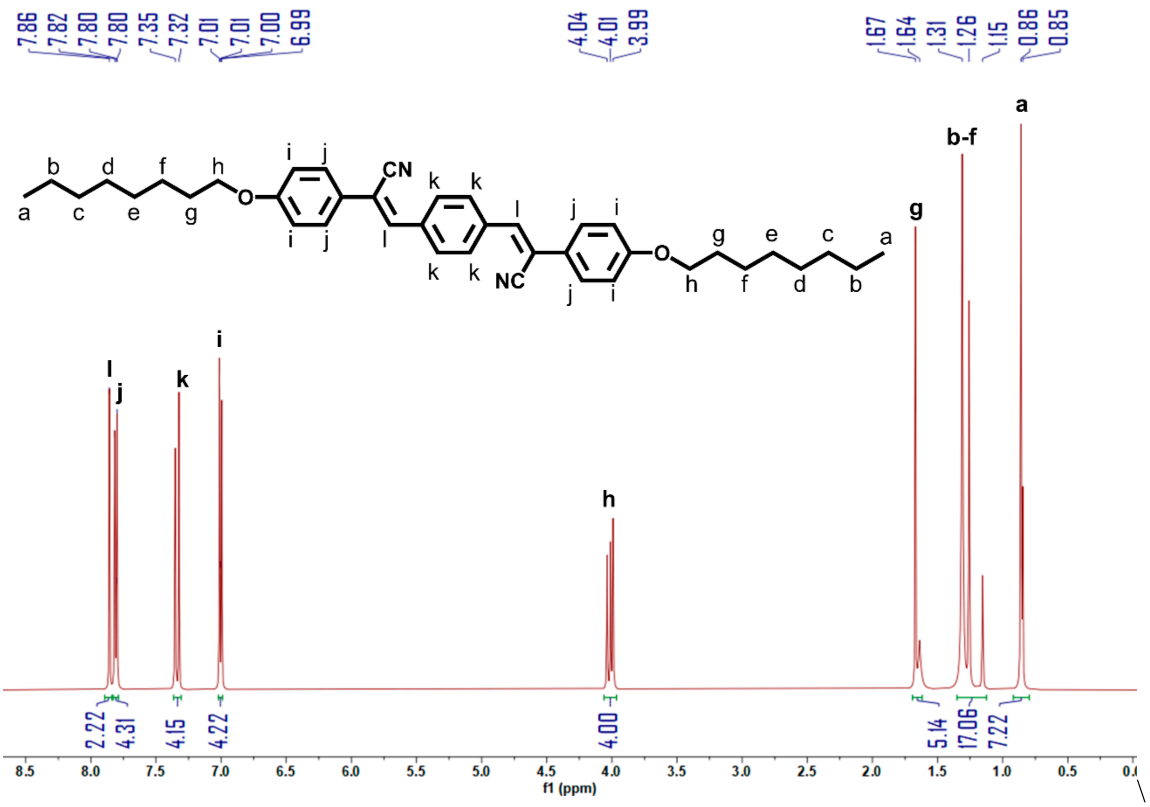

Figure S2. ^{1}H NMR spectrum of DC8 in $CDCl_3$.

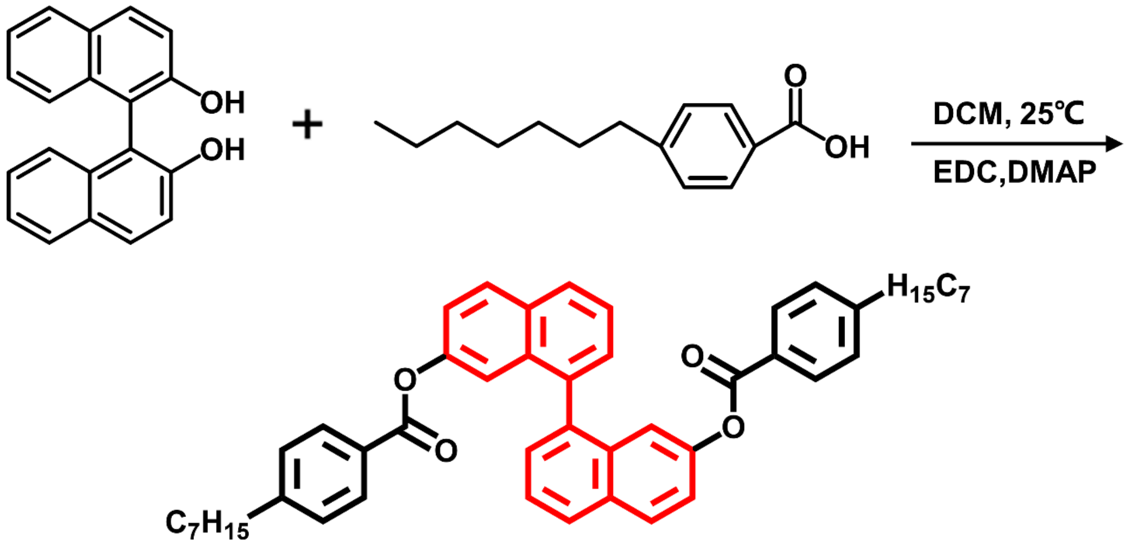

Figure S3. Synthetic route of chiral dopant

Binaphthalenediphenol (0.92 g, 3.4 mmol), heptylbenzoic acid (1.54 g, 7.0 mmol), 4-dimethylaminopyridine (0.1 g, 0.8 mmol), 1-(3-Dimethylaminopropyl)-3-ethylcarbodiimide hydro (1

g, 6.8 mmol) were added in dichloromethane (40 mL). This mixture was reacted at room temperature for 24 hours. After cooling to room temperature, the solution was filtered and then purified by column chromatography (DCM: PE 6 : 1). After the reagent was removed by rotary evaporation, a colorless and transparent liquid was obtained (1.6 g, 65%). ^{1}H NMR (600 MHz, Chloroform-d) δ 7.98 (d, J = 8.9 Hz, 2H), 7.91 (d, J = 8.2 Hz, 2H), 7.64 – 7.57 (m, 6H), 7.44 (dd, J = 15.6, 7.9 Hz, 4H), 7.33 (dd, J = 10.0, 6.6 Hz, 2H), 7.08 (d, J = 8.1 Hz, 4H), 2.62 – 2.54 (m, 4H), 1.57 (s, 4H), 1.29 (s, 16H), 0.90 (t, J = 6.9 Hz, 6H).

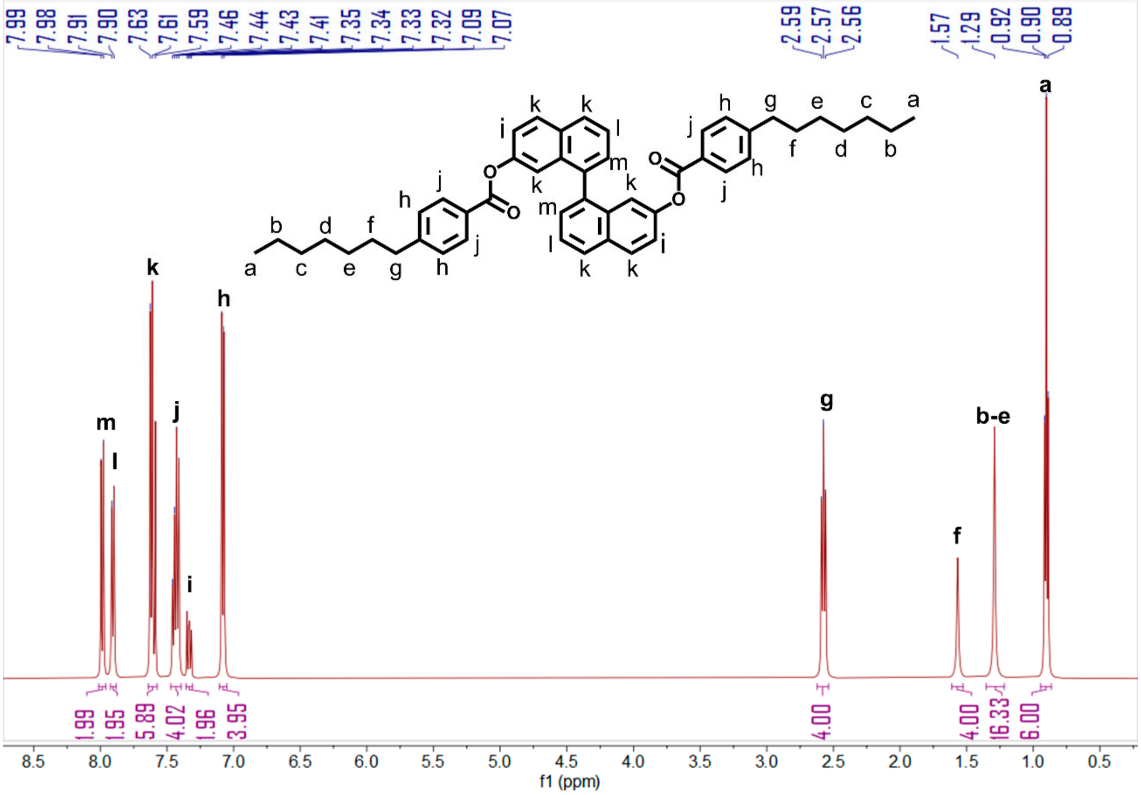

Figure S4. ^{1}H NMR spectrum of M in $CDCl_3$.

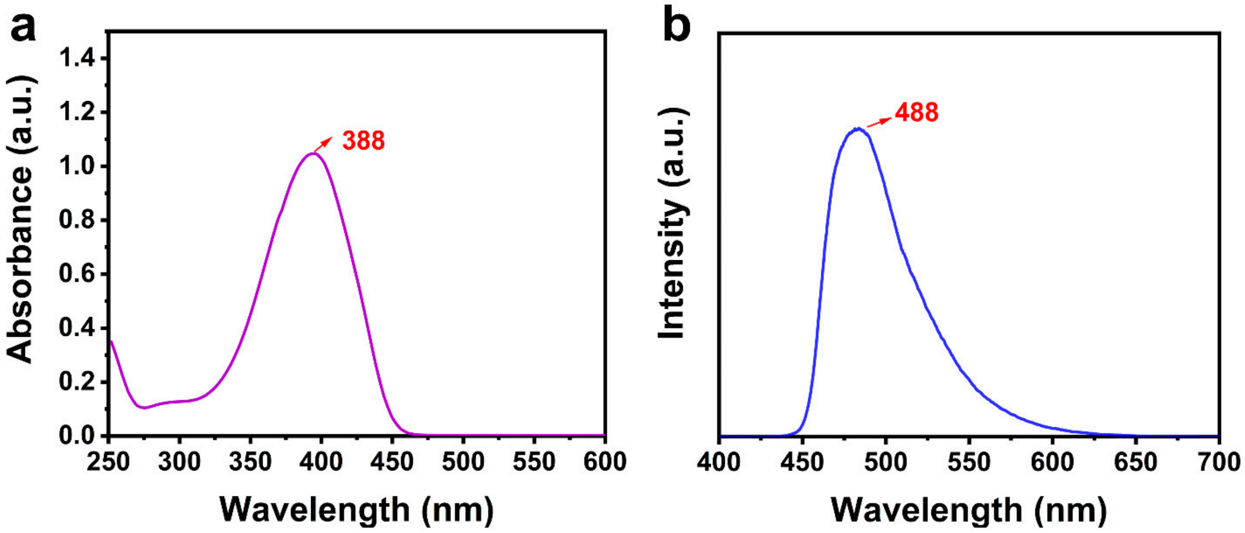

Figure S5. (a) The UV-vis spectrum of DC8 in THF (10^{-5}). The absorption maximum is 388 nm. (b) The photoluminescence (PL) spectra of DC8 in THF (10^{-5}). The emission maximum is 488 nm at the excitation wavelength of 388 nm.

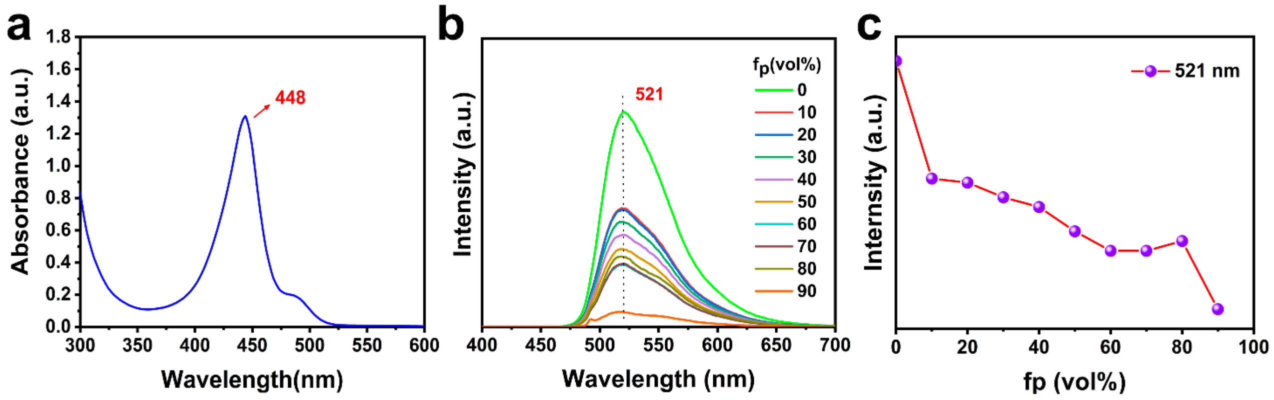

Figure S6. (a) The UV-vis spectrum of fluorescein in EtOH (10^{-5}). The absorption maximum is 448 nm. (b) The photoluminescence (PL) spectra of fluorescein in EtOH and EtOH/PE mixtures (10^{-5}). The excitation wavelength is 448 nm. (c) Diagram of the emission intensity at 521 nm versus PE fraction for fluorescein in EtOH and EtOH/PE mixtures.

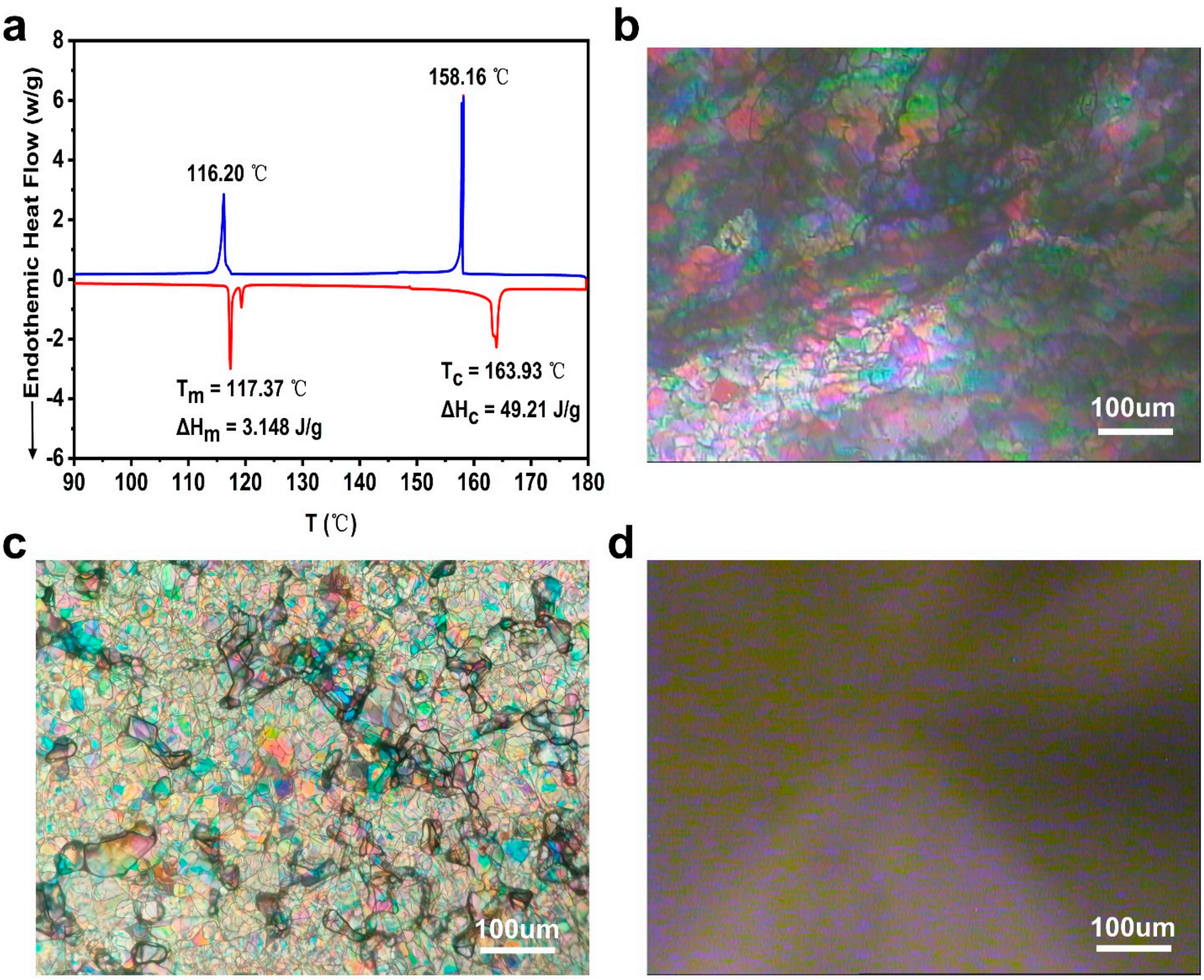

Figure S7. (a) DSC thermograms of the first heating cycle (red line) and first cooling cycle (blue line) cycle of DC8. (b) The POM photograph heating DC8 to 100 ºC. (c) The POM photograph heating DC8 to 140 ºC. (d) The POM photograph heating DC8 to 165 ºC.

Table S1 Fluorescent cholesteric phase liquid crystal system

	SLC-1717	S811	M	DC8
1	87.75 wt%	8 wt%	4 wt%	0.25 wt%
2	84.75 wt%	10 wt%	5 wt%	0.25 wt%
3	81.75 wt%	12 wt%	6 wt%	0.25 wt%
4	88 wt%	8 wt%	4 wt%	0

Preparation of the standard curve

The process of establishing the standard curve is shown in Figure S8a: Take channel C as an

example. Firstly, ten special x (x = 10, x = 20, x = 30 .... x = 100) values were picked and used CoreDrawr software to plot them as data that can be recognized by the inkjet printer. Then 10 circles with a radius of 8 cm were printed at the substrate of weighed using the C channel of the inkjet printer. Finally, after the solvent evaporated completely, this substrate was weighed again and the Y value was calculated to obtain the relationship between Y and X. It is worth noting that where x is discrete and widely spaced and it must be fitted to obtain the corresponding relationship curve. After trying different fitting methods, the fit of the power function ($y = ax^b$) was finally determined, in which the R^2 value can reach above 0.99 (The R value represents the degree of matching between the curve data and the current fitting method, and the closer the R value is to 1, the closer it is to 1 means that the correlation between the current data and the fitted curve is greater).

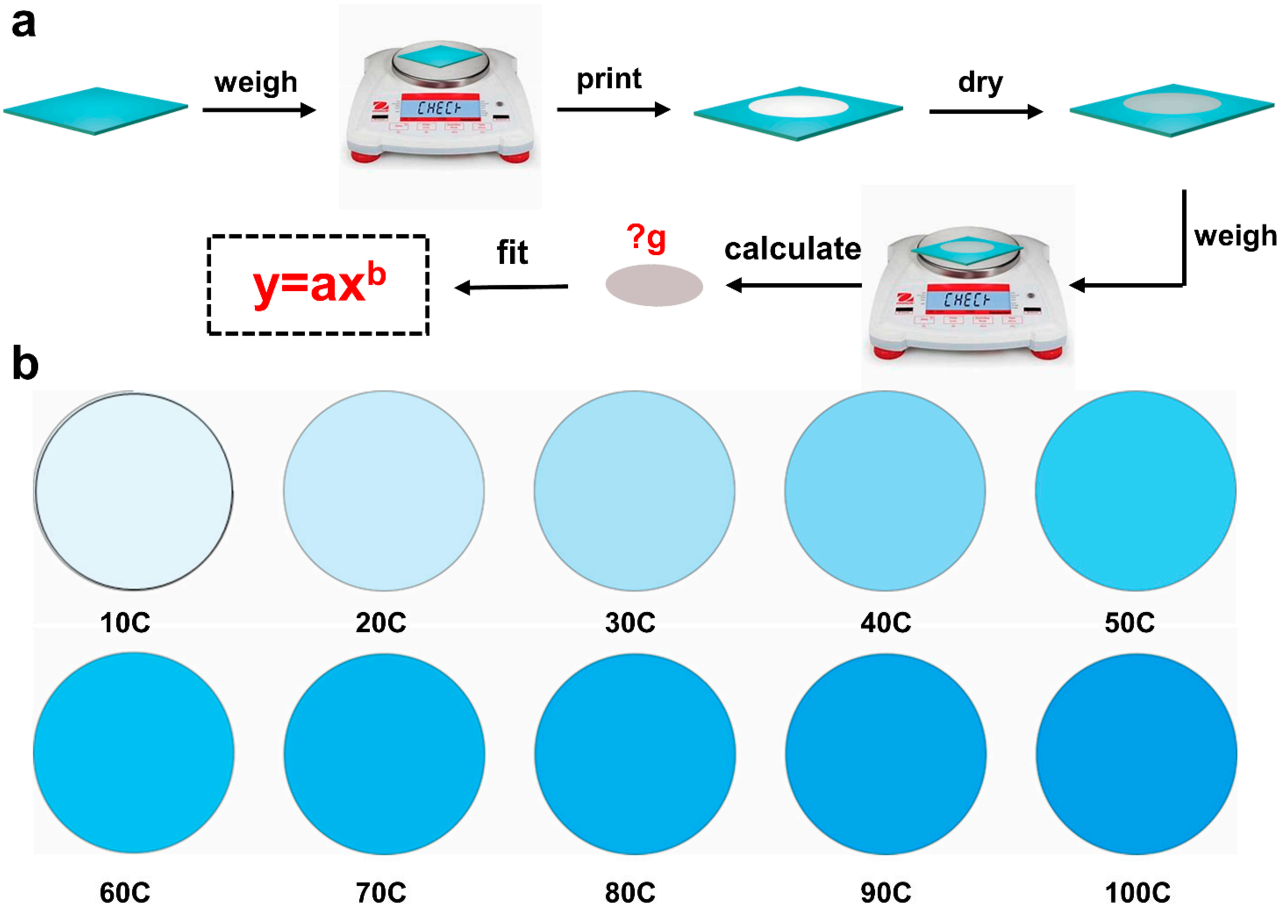

Figure S8. (a) Schematic of standard curve preparation. (b) Picture of the standard color block for printing.

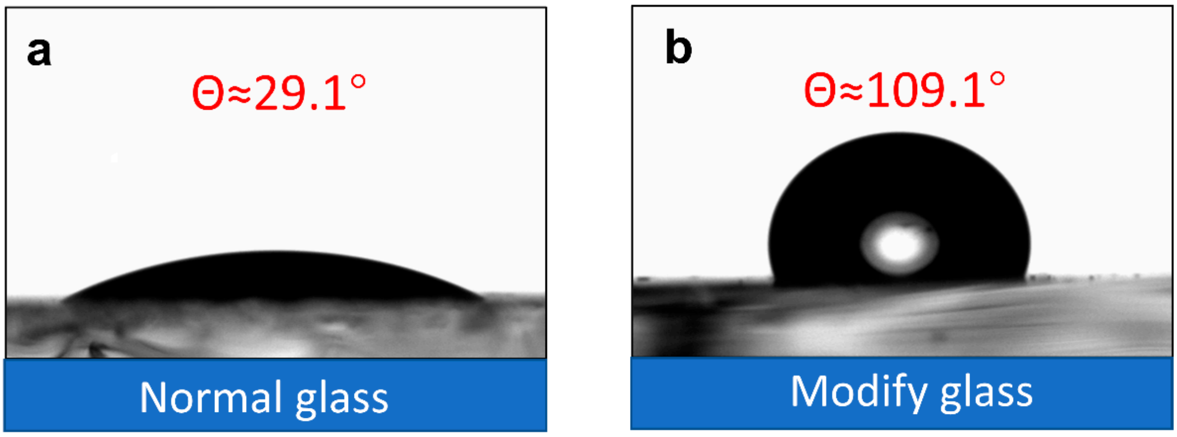

Figure S9. (a) The static contact angle of water on top of the normal glass (θ =2 9.1° for water). (b) The static contact angle of water on top of the glass modified Poly tetra fluoroethylene (θ = 109.1° for water).

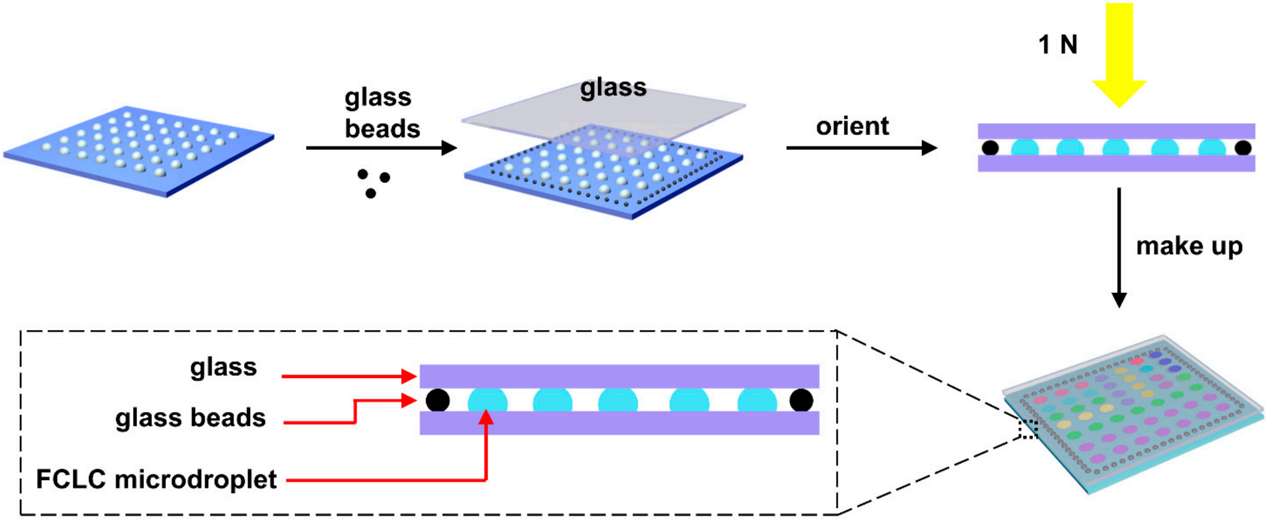

Figure S10. Schematic of LC microdroplet arrays orientation. The diameter size of glass beads is 5 μ m.

Table S2 The composition of LC microdroplets for patterning

	SLC-1717	S811	M	DC8
Green	85 wt%	10 wt%	5 wt%	0 %
F Green	84.8 wt%	10 wt%	5 wt%	0.2 wt%
Blue	82 wt%	12 wt%	6 wt%	0 %
F Blue	81.8 wt%	12 wt%	6 wt%	0.2 %
Gray	100 wt%	0 %	0 %	0 %
F Gray	99.8 wt%	0 %	0 %	0.2 wt%

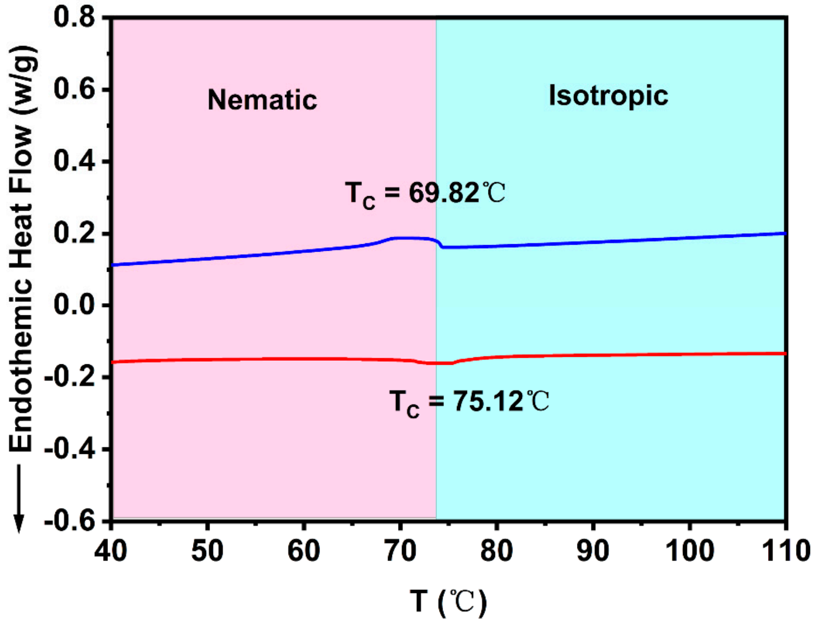

Figure S11. DSC curves of the fluorescent LC mixture (12 wt% S811, 6 wt% M, 0.2 wt% DC8, 81.8 wt% SLC-1717).
